# Subacute Combined Degeneration of the Spinal Cord: A Consequence of Recreational Nitrous Oxide Use

**DOI:** 10.7759/cureus.31936

**Published:** 2022-11-27

**Authors:** Tyler J Kingma, Soraya Bascoy, Muhammad D Altaf, Abhi Surampudy, Bilal Chaudhry

**Affiliations:** 1 Internal Medicine, Edward Via College of Osteopathic Medicine, Spartanburg, USA; 2 Internal Medicine-Pediatrics, Christiana Care Health System, Newark, USA; 3 Diagnostic Radiology, Christiana Care Health System, Newark, USA; 4 Internal Medicine, Christiana Care Health System, Newark, USA

**Keywords:** nitrous oxide toxicity, vitamin b12 supplementation, subacute combined degeneration of the spinal cord, functional vitamin b12 deficiency, causes of vitamin b12 deficiency, nitrous oxide abuse

## Abstract

A 21-year-old female presented to the hospital with worsening bilateral lower extremity weakness and sensory changes in the distal extremities following chronic nitrous oxide (N_2_O) abuse. Laboratory and radiographic results were suggestive of subacute combined degeneration of the upper cervical and thoracic spinal cord in the setting of a normal vitamin B12 level of 374 pg/mL with an elevation in methylmalonic acid to 1.14 mcmol/L. She was diagnosed with a relative B12 deficiency and treated with supplemental vitamin B12, resulting in an improvement in symptoms. This case highlights the importance of considering relative vitamin B12 deficiency as a diagnosis in the setting of nitrous oxide use, regardless of measured vitamin B12 level.

## Introduction

Nitrous oxide (N2O) has been used clinically for its sedative and analgesic properties in minor procedures since the mid-19th century [1]. However, given the advancements of modern anesthetics and the accumulating evidence of the adverse effects of nitrous oxide, its use clinically is declining. Despite the aforesaid, it is still used recreationally due to its euphoric effects, wide availability, and relatively low cost. It is commonly inhaled by breathing the fumes in close-range, concentrated settings via balloons or "whippets," which are small, steel, pressurized canisters filled with about eight grams of N2O [2]. Adverse effects of N2O intoxication can vary but commonly include short-lived euphoria, dizziness, slurred speech, drowsiness, and even loss of consciousness [3]. Although stored in excess in the liver, deficiency can occur through several mechanisms, including dietary insufficiency, malabsorption, and autoimmune pernicious anemia [4]. Nitrous oxide use is a less common but important cause of vitamin B12 deficiency that causes a functional deficiency regardless of serum level. In that case, B12 inactivation results in demyelination of the dorsal columns, spinocerebellar tracts, and/or lateral corticospinal tracts, leading to a range of neurologic deficits, from impaired proprioception to muscle weakness [5].

## Case presentation

We present the case of a 21-year-old female with no past medical history who presented to the hospital with one week of progressive bilateral leg weakness and numbness up to the level of the knee. This resulted in decreased stability and thus one to two falls at home per day. The patient also reported mild numbness in her hands to the level of the wrist but denied any weakness in those extremities. These symptoms were reportedly new, and she denied any form of trauma or inciting injury, headache, chest pain, changes in urinary or bowel habits, back pain, or dietary restrictions associated with these symptoms. Notably, the patient did admit to recreational nitrous oxide use of as much as 50 “whippets” (400 grams) a day to self-medicate for perceived depression.

On examination, her lower extremities had 0/5 strength in bilateral ankle dorsiflexion and plantarflexion with significant foot drop and 5/5 strength in bilateral hip flexion, knee flexion, and knee extension. The bilateral patellar reflex was 2/4, and the bilateral Achilles reflex was 0/4. There was intermittent sensation awareness in the posterior distal leg and the plantar and dorsal surfaces of the feet. Light touch sensation was intact in the proximal leg and anterior distal leg. Scattered ecchymoses at various stages of healing were noted on the bilateral lower legs to the level of the knee, presumably associated with her multiple falls at home. Upper extremity examination showed 4/5 strength in bilateral grip and 5/5 strength in bilateral elbow flexion/extension and wrist flexion/extension with intact upper extremity reflexes and pain/light touch sensation evaluated by pin prick and light brush throughout.

**Figure 1:**
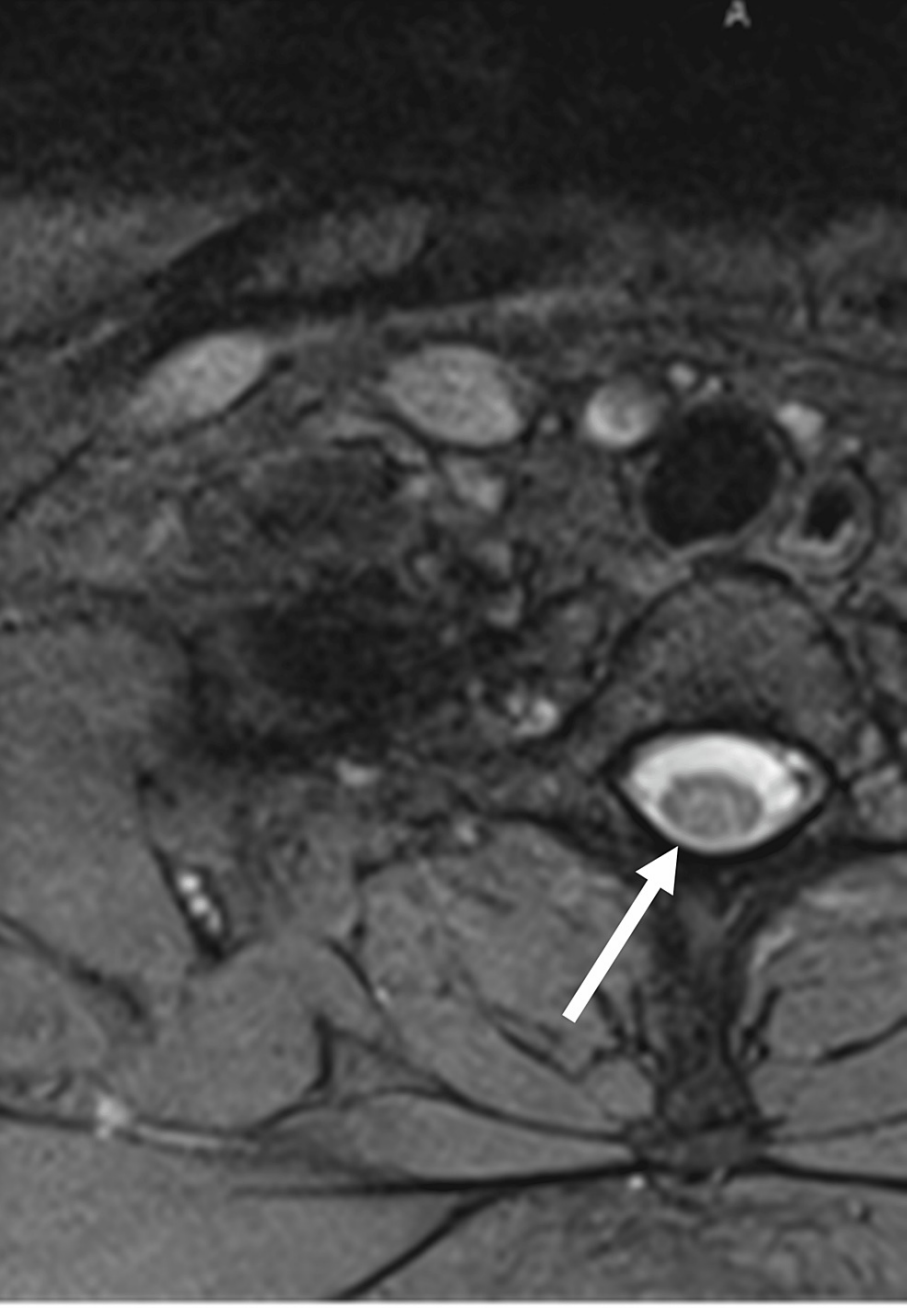
MRI of the cervical spine revealed an abnormal T2-hyperintense signal involving the dorsal column of the spinal cord.

Labs were remarkable for macrocytic anemia with a mean corpuscular volume (MCV) of 103.1 fL (ref: 80-100), a normal B12 level of 374 pg/mL (ref: 160-950), and an elevated MMA level of 1.14 mcmol/L (ref: 0.07-0.27). Heavy metals such as arsenic, lead, cadmium, and mercury were checked and found to all be at acceptable levels. MRI of the brain and cervical spine with and without contrast revealed an abnormal T2-hyperintense signal of the dorsal columns in the upper cervical spinal cord and upper thoracic spinal cord, as shown in Figure 1.

These findings were consistent with a relative B12 deficiency as the cause of subacute combined degeneration of the upper cervical and thoracic spinal cord regions of the spinal column in this patient. The patient received 1,000 mcg of intramuscular vitamin B12 three times per week and experienced an excellent response in terms of symptoms, eventually regaining full sensation in both upper and lower extremities within the first week of treatment. By two weeks, she had regained some ability to plantarflex bilateral feet with 3/5 strength; however, bilateral foot drop was sustained with 0/5 strength in foot dorsiflexion. The recommendation for continued treatment with supplemental vitamin B12 for an additional three weeks was made, but the patient ultimately signed out against medical advice.

## Discussion

The interaction between B12 and nitrous oxide was first evaluated by Banks, Henderson, and Pratt in 1968 and further evaluated by Nunn in 1987. They found that nitrous oxide converted cobalt from a monovalent to a bivalent form, restricting its use downstream as a cofactor in the conversion of methylmalonic acid (MMA) to succinyl-coenzyme A (succinyl-CoA) and the conversion of homocysteine (HC) to methionine [6-7]. The diagnosis of vitamin B12 deficiency is typically defined as levels less than 200 pg/mL, though there has been a lack of association between serum levels and diagnosis [8]. Thus, it has been suggested that a functional B12 deficiency can occur at any serum level due to alterations at the cellular level [9]. As such, methylmalonic acid and homocysteine levels have been shown to be significantly more sensitive for diagnosis [10]. Though not required, diagnosis can be supported by magnetic resonance imaging of the cervical spine, usually showing regions of T2 hyperintensity in the dorsal and lateral columns suggestive of the demyelination seen in subacute combined degeneration (SCD) [11]. 

Recognition and exclusion of possible alternative diagnoses such as transverse myelitis, infectious myelopathy, and copper deficiency are critical to expeditious treatment. Transverse myelitis could present similarly to SCD, though it has some key characteristics that make it less likely in this case. In transverse myelitis, demyelination is often limited to just one or two spinal levels, and demyelination does not preferentially involve the dorsal columns [12]. In our case, imaging revealed demyelination of the upper cervical and thoracic spinal cord, making this diagnosis less likely. Infectious myelopathy such as human immunodeficiency virus (HIV) or acquired immunodeficiency syndrome (AIDS)-associated vacuolar myelopathy should also be considered in this presentation. Symmetric involvement of the posterolateral columns would be expected with this condition, with similar findings on imaging [12]. Our patient has no history of HIV, opportunistic infections, or malignancy, making this diagnosis even less likely. A final alternative diagnosis of copper deficiency has been found to cause hyperintensity of the posterior columns of the cervical cord [12]. While there was some degree of symptom improvement with supplemental vitamin B12, making copper deficiency less likely, it cannot be completely ruled out given the lack of complete symptom resolution. Though it is common not to achieve complete symptom remission, a follow-up on this patient’s serum copper and ceruloplasmin levels is warranted to rule out copper deficiency as a cause of the remaining symptoms. Despite there being no standardized treatment, typical therapy includes an initial combination of daily intramuscular or oral B12 supplementation at 1000-2000 micrograms, followed by weekly supplementations. Recovery of neurologic symptoms is often slow, and return to the neurological baseline is variable despite the usual correction of serum methylmalonic levels. [13-14]. Electromyography could be considered if neurologic symptoms are not resolving.

## Conclusions

Recreational nitrous oxide abuse can lead to varying levels of reversible and/or irreversible degeneration of the spinal cord due to alterations in the metabolism of vitamin B12. Providers should consider this as a diagnosis in patients presenting with unexplained neurologic symptoms, regardless of the B12 level, in the setting of elevated methylmalonic acid (MMA) and homocysteine (HC). For the safety of those using N2O recreationally, these severe consequences should be better communicated, in addition to further evaluation of the continued legality of the drug. If the recreational use of N2O continues, it will be critical to establish SCD treatment guidelines to provide consistency in therapy and potentially improve outcomes.
